# Crisis Team Management in a Scarce Resource Setting: Angkor Hospital for Children in Siem Reap, Cambodia

**DOI:** 10.3389/fpubh.2017.00154

**Published:** 2017-07-05

**Authors:** Richard Alynn Henker, Hiroko Henker, Hor Eng, John O’Donnell, Tachawan Jirativanont

**Affiliations:** ^1^Department of Nurse Anesthesia, School of Nursing, University of Pittsburgh, Pittsburgh, PA, United States; ^2^Graduate School of Public Health, University of Pittsburgh, Pittsburgh, PA, United States; ^3^Angkor Hospital for Children, Siem Reap, Cambodia; ^4^Faculty of Medicine, Department of Anesthesiology, Siriraj Hospital, Mahidol University, Bangkok, Thailand

**Keywords:** simulation, crisis team management, non-technical skills, cambodia, health volunteers overseas

## Abstract

**Introduction:**

A crisis team management (CTM) simulation course was developed by volunteers from Health Volunteers Overseas for physicians and nurses at Angkor Hospital for Children (AHC) in Siem Reap, Cambodia. The framework for the course was adapted from crisis resource management (1, 2), crisis team training (3), and TeamSTEPPs© models (4). The CTM course focused on teaching physicians and nurses on the development of team performance knowledge, skills, and attitudes. Challenges to providing this course at AHC included availability of simulation equipment, cultural differences in learning, and language barriers. The purpose of this project was to evaluate the impact of a CTM simulation course at AHC on attitudes and perceptions of participants on concepts related to team performance.

**Methods:**

Each of the CTM courses consisted of three lectures, including team performance concepts, communication, and debriefing followed by rotation through four simulation scenarios. The evaluation instrument used to evaluate the AHC CTM course was developed for Cambodian staff at AHC based on TeamSTEPPs© instruments evaluating attitude and perceptions of team performance (5). CTM team performance concepts included in lectures, debriefing sessions, and the evaluation instrument were: team structure, leadership, situation monitoring, mutual support, and communication. The Wilcoxon signed-rank test was used to analyze pre- and post-test paired data from participants in the course.

**Results:**

Of the 54 participants completing the three CTM courses at AHC, 27 were nurses, 6 were anesthetists, and 21 were physicians. Attitude and perception scores were found to significantly improve (*p* < 0.05) for team structure, leadership, situation monitoring, and communication. Team performance areas that improved the most were: discussion of team performance, communication, and exchange of information.

**Conclusion:**

Teaching of non-technical skills can be effective in a setting with scarce resources in a Southeastern Asian country.

## Introduction

The development of non-technical skills has been shown to improve patient outcomes during crisis situations in high acuity clinical settings (6, 7). Team performance is a concern in health-care settings around the world, not just for clinicians in countries that have resources to provide simulation education using computerized mannequins (often termed high-fidelity simulators) and other technology-intense equipment. Simulation technology is expensive and requires technical support that is often not available in low- and middle-income countries. Improving team performance is a priority for health-care providers in low- and middle-income countries that realize the impact of non-technical skills on patient outcomes. Angkor Hospital for Children (AHC) in Siem Reap, Cambodia, provides health care for children in one of the poorer areas of Cambodia. AHC focuses on improving clinical care by advancing the education of the Cambodian nurses and physicians at AHC (8).

Crisis team management (CTM) focuses on team performance skills during an emergent situation that occurs when a patient has a clinical event that requires immediate intervention. Team work during a crisis situation impacts outcome no matter the setting (6, 7). The focus of this article is to describe the implementation and evaluation of a CTM course supported by volunteers from Health Volunteers Overseas (HVO) in a setting with scarce resources and strong cultural differences from Western settings using simulation education to improve non-technical skills.

## Angkor Hospital for Children

Angkor Hospital for Children is a non-government organization (NGO) hospital that provides free high-quality compassionate care to Cambodian children. The outpatient department at AHC sees 400–500 patients per day. The inpatient department includes a 40-bed inpatient unit, a 10-bed low acuity unit, a 11-bed surgical unit, and a 16-bed combined emergency/ICU unit. Surgical procedures are performed in a main operating theater, minor procedure room, and ophthalmology operating theater. An AHC satellite provides pediatric and neonatal service located at a government referral hospital in Sotnikum, 35 km from AHC. Travel is a major obstacle for many families to have their children receive health care in rural Cambodia, the satellite hospital provides access to care for those families that cannot afford to travel to or stay in Siem Reap. Although AHC is an NGO, funding from the World Health Organization and USAID provides support for programs provided in conjunction with the Ministry of Health (9). Support for the CTM course was provided by HVO volunteers and Faculty of Medicine, Siriraj Hospital, Mahidol University, Bangkok, Thailand.

Angkor Hospital for Children has been designated as a teaching hospital by the Ministry of Health. Pediatric clinical rotations are provided for nursing students from all five government schools of nursing. Medical students from Phnom Penh and international sites participate in clinical educational experiences provided by AHC physicians and nurses. A pediatric residency program was established at AHC in 2003. Educational programs such as Advanced Pediatric Life Support (APLS) were initially provided by an Australian team of certified APLS instructors but train the trainer courses led to the development of an independent AHC-delivered Khmer language APLS course, which mirrors the international one. Training of physicians and nurses when using APLS focuses more on application of protocols for treatment in emergency situation, whereas CTM focuses on team performance and communication during emergency situations.

Health Volunteers Overseas has been sending volunteers to AHC to support their Nurse Anesthesia program since 2004. The guiding principles of HVO are to improve care by teaching health-care providers in resource scarce settings (10). In addition, HVO volunteers teach existing health-care providers how to educate others. The CTM course follows the HVO model. The role of HVO volunteers expanded when the executive director of AHC in 2012 identified a need for focus on clinical communication skills. In collaboration with the staff at AHC, the CTM course focusing on team performance was adapted to meet the needs to improve clinical communication. The development of a CTM train the trainer course has led to direction of simulation scenarios and debriefing by Khmer nurses and physicians.

The CTM course was first taught by HVO volunteers from University of Pittsburgh and faculty from the Department of Anesthesiology, Siriraj Hospital, Mahidol University School of Medicine, in August of 2015. The goal of the CTM project is to develop instructors at AHC to assume the teaching of the CTM course in Khmer. Train the trainer courses have been conducted in October 2016 and March of 2017. Additional train the trainer courses have been scheduled in 2017 to reinforce previous training of instructors and to train new instructors as needed (see Table 1).

**Table 1:** Crisis team management (CTM) courses offered and planned for Angkor Hospital for Children.

Date of CTM course	Instructor trainer course	Health Volunteers Overseas volunteer instructors	International instructors
August 2015	Not offered	2	5
March 2016	Not offered	3	0
October 2016	6	3	0
March 2017	6	2	2
July 2017	Yes	2	2
October 2017	Yes	0	2

## Does High-Fidelity Simulation Education Improve Patient Outcomes?

Simulation education has been found to be beneficial in improving outcomes in the clinical setting. In a meta-analysis of 609 studies done by Cook and colleagues (11), technology-enhanced simulation training in health professionals was found to have a large effect for participants in terms of knowledge, skills, and behaviors and a moderate effect on patient outcomes.

Although simulation has been shown to be effective, many assume that high-fidelity simulation is more effective than low-fidelity simulation. The cost of the technology to support simulation can quickly become prohibitive in a scare resource setting such as AHC. High-technology simulators can cost over $60,000 (personal communication, John O’Donnell). In a meta-analysis, Norman and colleagues (12) compared the effectiveness of high-fidelity versus low-fidelity simulation teaching. Outcomes of the 24 studies included in the meta-analysis were auscultation skills, surgical techniques, and complex management skills. There was no difference in high-fidelity versus low-fidelity methods of teaching on outcomes. Norman and colleagues (12) suggest that more complex skills do not require more complex simulators. Although high-fidelity simulation methods were considered at AHC, cost could not justify the purchase of high-tech simulators. Thus, low-fidelity simulation was used to support the teaching of non-technical skills at AHC.

Authenticity is considered a key concept when using simulation (12). Is the simulation setting close to real life? Norman and colleagues (12) describe fidelity to support the authenticity of a simulation setting in two ways. Engineering fidelity has been described by "does the setting look real?" Psychological fidelity has been described as, "does the setting elicit the required behaviors?" A dedicated simulation laboratory with a high-tech simulator is not required to improve competencies related to non-technical skills. At AHC, two or three classrooms, depending upon the size of the class, were used to run simulation scenarios and conduct debriefing sessions.

## How Should We Teach Non-Technical Skills?

Education for high acuity crisis situations in clinical setting is often associated with the development of task-oriented education that focuses on skills and knowledge. Advanced Cardiac Life support is an example of a course that provides this knowledge and skills with some focus on team performance (13–15). As health-care providers learn more about the importance of team performance during acute care crisis situation, there is increased demand for team performance and non-technical skill training. The development of non-technical skills is associated with improved knowledge, confidence, and skills among health-care providers (16). Additionally some studies suggest that good team performance is associated with improved patient outcomes (6, 7).

## What Crisis Team Performance Models were Used at AHC?

The CTM course at AHC was developed based upon various models for crisis team performance also known as non-technical skills. Crisis Team Training (CTT) developed by DeVita et al. (3) at the Winter Institute for Simulation Education and Research focused on completion of team tasks, and directed communication. A key concept in the CTT course was a flattening of the power hierarchy (doctor versus nurse) that promoted communication between team members. Gaba (2) developed an Anesthesia Crisis Resource Management course that utilized concepts used in the air-line industry. The course focused on communication including, situation, background, assessment and recommendations (SBAR) used during handoffs. SBAR has been incorporated into CTM at AHC. TeamSTEPPs© (4) provided concepts that were incorporated during teaching of non-technical skills, team structure, leadership, mutual support, situational awareness, and communication.

## How is Learning Different in Cambodia?

Communication during the CTM course is an important concept for the success of team performance. CTM courses at AHC have been taught in English. Although participants speak English relatively well, the language typically used during crisis events at AHC is Khmer. The first three times the CTM course was offered in August 2015, March 2016, and October of 2016, the course was taught in English. Lectures for the fourth course in March of 2017, were taught in English but scenarios and debriefing were conducted mostly in Khmer. Instructor trainers directed scenarios and led debriefing. HVO volunteers and international faculty initially directed scenarios and led debriefing sessions. Future courses to be offered in 2017 will be led by AHC faculty, and volunteer faculty will serve as consultants for the CTM course. See Table 1 for a timeline of the CTM course that have been offered and are planned. The goal for the CTM course is that it will be coordinated and taught by AHC faculty with little support by HVO volunteers and international faculty.

Learning styles by students in Cambodia are rapidly changing from the use of a more traditional classroom environment to frequent use of the internet and other less traditional methods of learning. In the past, the learning environment focused more on memorization and less on critical thinking (17). Students feared asking questions in class or answering questions posed by the instructor incorrectly (18). Students in Asian cultures were more likely to ask questions during individual meetings with instructors rather than group sessions. Facilitating classroom discussion and using less traditional teaching methods is strongly emphasized at AHC. The goal at AHC is to have considerable discussion and promote participation in the classroom and less pure lecture style of teaching. During the first CTM course, discussion during debriefing sessions were very lively and often went past the allotted time due to the rich discussion by the more senior participants enrolled in the first course.

Advanced Pediatric Life Support is mandatory at AHC and for pediatricians in countries such as the UK. The knowledge from APLS is known to saves lives. However, APLS focuses on the individual’s performance during scenarios—particularly memorization and application of evidence-based protocols. Participants called on to assist in a scenario may only do what the individual being tested asks of them and cannot contribute their own knowledge or observations. As such, it does not focus on team performance skills. During the CTM course, Khmer instructor trainers would sometimes focus upon discussion of the APLS protocol alone with less emphasis on team performance concepts. HVO volunteer instructors would assist by refocusing debriefing on concepts related to team performance. Gaining expertise with protocols is much more comfortable with learners and Khmer instructor trainers well versed in APLS than applying team performance concepts in a crisis situation. The introduction of non-technical skills that is new to health-care providers at AHC was difficult at times and required critical thinking skills but by the end of the course participants seemed to be enthusiastic about learning related to team performance skills.

## Methods

Institutional Review Board approval was provided for this project by AHC. Physicians and nurses employed at AHC and its Satellite Clinic in Sotnikum participated in three CTM courses offered in August of 2015, March of 2016, and October of 2016. Instructor trainer courses were offered in October of 2016 and March of 2017.

### Faculty

Instructors for the course were from Faculty of Medicine, Siriraj Hospital, Mahidol University, Bangkok, Thailand, and Graduate School of Public Health and School of Nursing at the University of Pittsburgh, USA. In addition to stating their role and unit at AHC, participants completed a pre- and post-course questionnaire regarding perceptions and attitudes associated with team performance.

### Components of the Course

The 8-h CTM course comprised of three lectures and rotation through four simulation scenarios. Course concepts were based on the Crisis Resource Management model (2) CTT (3) and TeamSTEPPs© (4). Development of scenarios, debriefing points and evaluation were based on the concepts of (1) team structure, (2) leadership, (3) situational awareness, (4) mutual support, and (5) communication. Lectures covered CTM principles, communication skills, and debriefing. Initially, lectures, simulation scenarios, and debriefing sessions were conducted in English. Simulation scenarios were developed and directed by simulation instructors from Thailand (Tachawan Jirativanont), US (Richard Alynn Henker), and Japan (Hiroko Henker).

Crisis scenarios included anaphylaxis, hemorrhagic shock, postoperative bleeding after tonsillectomy, hypoxemia, and opioid-induced respiratory depression. A scenario template from Siriraj Hospital included setup instructions, flow of the scenario, and debriefing points. Although there may be some discussion during the CTM debriefings about appropriate treatment, the focus of debriefing was on concepts related to team performance. Instructors for the third courses included nurses and physicians from AHC who had been enrolled in the train the trainer course. Confederates, playing the roles of patient family members for simulation scenarios included nurse anesthetists from Siriraj Hospital and nurse anesthesia students from the University of Pittsburgh, School of Nursing.

Low-tech pediatric manikins that could be intubated were available at AHC for the CTM course. These manikins were typically used for the APLS course at AHC and do not have the technological capabilities of manikins used in simulation lab settings in the US. Ipads (Apple, Cupertino, CA, USA) were configured as physiologic monitors to provide heart rate, oxygen saturation, blood pressure, and respiratory rate during simulation scenarios. The app used to configure the Ipads as physiologic monitors was Sim Mon version 1.5.3 (Castle + Andersen Aps, Denmark). Airway equipment including oral airways, endotracheal tubes, and laryngoscopes were available from the APLS course at AHC. Intravenous infusions were attached to manikins and medications were labeled and available.

### Debriefing Techniques

Debriefing methods used for the CTM course were based upon experiential learning by Kolb (19) and focused on providing a concrete experience with an emphasis on reflective observation. Other models influencing our debriefing approach included (1) plus delta and (2) gather, analyze and summarize (20). During the train the trainer course, debriefing concepts were reviewed based on categories from the Debriefing Assessment for Simulation in Healthcare instrument emphasizing: (1) having participants describe their thoughts on how they performed, i.e., reflection, (2) providing a structured debriefing format including a review of the concepts of team performance, and (3) having participants summarize team performance by describing aspects of the simulation scenario that went well and those that needed improvement (21). These principles were used to guide debriefing of CTM participants immediately after scenarios. Duration of debriefing sessions was 20–30 min.

### Course Evaluation

Attitudes and perceptions of team performance including team structure, leadership, mutual support, situational awareness, and communication were evaluated in participants before and after the course. The 18-item evaluation instrument with responses of 1 (agree) to 5 (disagree) was used to evaluate the AHC CTM course concepts of team performance. The instrument was developed based on TeamSTEPPs© perception and attitude instruments by BunRum Ly and R. Henker (5). The instrument used for the course was in English at a level appropriate for Cambodia staff at AHC. Subscales in the instrument match the concepts used when teaching the course, i.e., team structure, leadership, situation monitoring, mutual support, and communication. The instrument focused on attitudes and perceptions of course participants. Data for statistical analysis were entered into SPSS Version 24 (2016). Pre and post scores were compared using Wilcoxon signed-rank test for participants in the simulation workshop.

## Results

Twenty-seven nurses, 6 anesthetists, and 21 physicians participated in three CTM courses offered August of 2015, March of 2016, and October of 2016. Pre- and post-course questionnaire data were collected on 54 course participants. Of the 54, 21 were from the operating theater and 13 were from the emergency department/intensive care unit. The remaining participants were from a wide variety of units at AHC including the inpatient department, outpatient department, satellite, eye clinic, and external programs.

Comparison of pre and post perception and attitude scores demonstrated statistically significant improvement for team structure (*p* = 0.00), leadership (*p* = 0.00), situation monitoring (*p* = 0.00), and communication (*p* = 0.00). Although not statistically significant, the *p*-value for mutual support was *p* = 0.09. See Tables 2 and 3 for changes in by team performance concept category.

**Table 2:** Wilcoxon signed-ranks test results by team performance category.

Team performance	Post minus pre-negative ranks	Post minus pre-positive ranks	Ties	*p*-Value
Team structure	26	7	18	0.00
Leadership	31	6	16	0.00
Situation monitoring	26	6	20	0.00
Mutual support	23	12	16	0.08
Communication	30	8	15	0.00

**Table 3:** Average scores by team performance category.

Team performance	Pre-course mean score	Post-course mean score	Paired differences	Paired difference SD
Team structure	6.47	5.47	0.71	1.7
Leadership	6.51	5.53	0.98	1.4
Situation monitoring	5.35	4.56	0.79	1.4
Mutual support	6.55	6.04	0.51	2.0
Communication	5.74	4.13	1.60	2.7

The survey question areas that had the greatest and least change for each team performance concept topic are listed in Table 4. The areas associated with the greatest changes in the perception and attitude scores; (1) discussion of team performance, (2) exchange of information by the team, and, (3) good communication is associated with better team performance.

**Table 4:** Survey questions with the greatest and least change for each team performance category.

Team performance concepts	Significance by team performance concept	Pre-post negative ranks	Pre-post positive ranks	Ties	*p*-Value
**Team structure**					
Team goals more important than individual goals	Greatest change	7	0	46	0.01
Staff share information to improve team performance	Least change	16	7	29	0.08
**Leadership**					
Leader provides opportunity to discuss team performance	Greatest change	25	3	25	0.00
Leader views mistakes as learning opportunity	Least change	14	7	32	0.13
**Situation monitoring**					
Staff exchange of important information	Greatest change	22	3	27	0.00
Monitor stress of team members and assist if needed	Least change	14	8	31	0.13
**Mutual support**					
Help other team members is part of good team performance	Greatest change	15	4	34	0.03
Communicate during stressful times in positive way	Least change	14	11	26	0.92
**Communication**					
Poor communication common cause of error	Greatest change	21	3	29	0.00
Staff share information in timely manner	Least change	18	8	27	0.28

## Discussion

Participant evaluations at the end of the course indicated that the scenarios were realistic and that it was important for them to reflect on their performance during debriefing. Participants would have liked readings assigned prior to the course to help prepare. Participants also indicated that they would prefer less lecture and more scenarios. The course offered in March of 2017 included six scenarios instead of four that had been previously used in prior courses. Additional scenarios for the March 2017 course were developed during the instructor trainer course that was held prior to the CTM course. Khmer instructors noted that a team work exercise early in the course engaged learners. Khmer instructors also noted that learning took place despite the low tech manikins used to support the course. International faculty noted that organizing the course was difficult due to instructors being from the US and Thailand.

Similar simulation courses have been offered in countries such as Ghana. In the Bulletin of the American College of Surgeons (22) the development of a simulation-based Advanced Trauma Operative Management course was reported. It was noted that the success of the course required substantial support from Johnson and Johnson for course implementation and in resourcing the simulation center. “Is the cost of a simulation center worth the benefits?” is a frequent question asked in low- and middle-income countries with scarce resources; although, the time commitments of instructors and participants needs to be considered in the cost.

### Partnerships

The partnership between the HVO nurse anesthesia program and AHC has been in place since 2004. This relationship has progressed from clinical teaching of basic skills for the anesthetists at AHC to the development of a CTM course to teach team performance knowledge and skills to nurses and physicians. The focus of initial volunteers was to assist AHC staff with support for the day-to-day function of the operating theater. As the partnership of HVO and AHC has grown, plans for volunteers have become more sophisticated with well-developed goals for projects. The development of the CTM course with specific goals and objectives is an example of the growth of the partnership between AHC and HVO and well-planned use of volunteers by AHC.

#### Sustainability

The model of educational development used by HVO contributed to the successful implementation and sustainability of this project. The need for improved clinical communication was identified by AHC and conveyed to HVO volunteers. A CTM Course that had been developed for Siriraj Hospital was adapted to AHC. Initially participants were taught in the CTM course that was developed with input from AHC educators. The next stage was the development of instructors from AHC to continue to have this course offered with little assistance from international and HVO volunteers.

#### Capacity Building

Although this specific project focuses on improving the quality of care at AHC through the development of the CTM course, it should be noted that the teaching by HVO nurse anesthesia volunteers has led to an increase in the anesthesia work force. The number of anesthesia providers at AHC has increased from three in 2006 to six in 2017. This increase in the number of anesthesia providers has led to opening of a minor procedure room and ophthalmology operating theater. In addition, HVO volunteers have assisted with the coordination of clinical experiences for AHC anesthesia providers at the National Pediatric Hospital in Phenom Penh, Siriraj Hospital in Bangkok and UPMC-Children’s Hospital of Pittsburgh in the US. Currently, the nurse anesthetists at AHC are not only teaching in the CTM course but are also providing a clinical rotation for nurses from Lao Friends Hospital for Children that are learning to be nurse anesthetists.

## Conclusion

The development of a CTM course at AHC provides an example of how to advance a sustainable education project that improves the quality of care in a resource scarce setting. The timing of this project was effective not only because of the need for improvement of clinical communication but also the ability of the AHC educators and administration to understand how to utilize HVO volunteers effectively. This project was driven by the HVO model to develop sustainable programs to educate health-care providers in a resource scarce environment then increase the likelihood of sustainability by developing a train the trainer course.

## Ethics Statement

This study was carried out in accordance with the recommendations of “Angkor Hospital for Children Institutional Review Board.” The protocol was approved by the “Angkor Hospital for Children Institutional Review Board.”

## Author Contributions

RH co-coordinated the course, developed scenarios, conducted the analysis, wrote the manuscript, and taught in the course. HH co-coordinated the course, developed simulation scenarios, assisted with the analysis, and taught in the course. TJ co-coordinated the course, developed simulation scenarios, and taught in the course. HE taught in the course and assisted with the development of the manuscript. JO assisted with analysis and development of the manuscript.

## Conflict of Interest Statement

The authors declare that the research was conducted in the absence of any commercial or financial relationships that could be construed as a potential conflict of interest.

## References

[1] Cheng A Donoghue A Gilfoyle E Eppich W. Simulation-based crisis resource management training for pediatric critical care medicine: a review for instructors. Pediatr Crit Care Med (2012) 13(2):197–203. 10.1097/PCC.0b013e3182192832 21499181

[2] Gaba DM Crisis resource management and teamwork training in anaesthesia. Br J Anaesth (2010) 105(1):3–6. 10.1093/bja/aeq124 20551023

[3] DeVita MA Schaefer J Lutz J Dongilli T Wang H Improving medical crisis team performance. Crit Care Med (2004) 32(Suppl):S61–5. 10.1097/01.ccm.0000110872.86812.1c 15043232

[4] Agency for Healthcare Research and Quality. About TeamSTEPPS. (2016). Available from: http://www.ahrq.gov/professionals/education/curriculum-tools/teamstepps/about-teamstepps/index.html 10.1080/15360280802537332 21923316

[5] Castner J. Validity and reliability of the brief TeamSTEPPS teamwork perceptions questionnaire. J Nurs Meas (2012) 20(3):186–98. 10.1891/1061-3749.20.3.186 23362556

[6] Baker VO Cuzzola R Knox C Liotta C Cornfield CS Tarkowski RD Teamwork education improves trauma team performance in undergraduate health professional students. J Educ Eval Health Prof (2015) 12:36. 10.3352/jeehp.2015.12.36 26101404 PMC4536358

[7] Riley W Davis S Miller K Hansen H Sainfort F Sweet R Didactic and simulation nontechnical skills team training to improve perinatal patient outcomes in a community hospital. Jt Comm J Qual Patient Saf (2011) 37(8):357–64. 10.1016/S1553-7250(11)37046-8 21874971

[8] Angkor Hospital for Children. Treatment Education, Prevention. (2015). Available from: http://angkorhospital.org/education/

[9] Henker R Prak M Koy V. Development and implementation of cornerstone documents to support nursing practice in Cambodia. Online J Issues Nurs (2015) 20(2):5. 10.3912/OJIN.Vol20No02Man05 26882424

[10] Overseas HV Health Volunteers Overseas Guiding Principles. (2017). Available from: https://hvousa.org/whoweare/our-mission/

[11] Cook DA Hatala R Brydges R Zendejas B Szostek JH Wang AT Technology-enhanced simulation for health professions education: a systematic review and meta-analysis. JAMA (2011) 306(9):978–88. 10.1001/jama.2011.1234 21900138

[12] Norman G Dore K Grierson L. The minimal relationship between simulation fidelity and transfer of learning. Med Educ (2012) 46(7):636–47. 10.1111/j.1365-2923.2012.04243.x 22616789

[13] Hoadley TA. Learning advanced cardiac life support: a comparison study of the effects of low- and high-fidelity simulation. Nurs Educ Perspect (2009) 30(2):91–5. 19476072

[14] Rodgers DL Bhanji F McKee BR. Written evaluation is not a predictor for skills performance in an advanced cardiovascular life support course. Resuscitation (2010) 81(4):453–6. 10.1016/j.resuscitation.2009.12.018 20117875

[15] Yang TM Kao Y Wang CT Chung MH Lin HJ Lin SJ ACLS training: comparison of physicians and nurses with teamwork-based high-fidelity simulation. Am J Emerg Med (2014) 32(9):1132–4. 10.1016/j.ajem.2014.05.027 24974370

[16] Watters C Reedy G Ross A Morgan NJ Handslip R Jaye P. Does interprofessional simulation increase self-efficacy: a comparative study. BMJ Open (2015) 5(1):e005472. 10.1136/bmjopen-2014-005472 25586366 PMC4298099

[17] Chung HS Dieckmann P Issenberg SB. It is time to consider cultural differences in debriefing. Simul Healthc (2013) 8(3):166–70. 10.1097/SIH.0b013e318291d9ef 23702587

[18] Park C Learning style preferences of Southeast Asian students. Urban Educ (2000) 35(3):245–68. 10.1177/0042085900353002

[19] Kolb D Experiential Learning: Experience as the Source of Learning and Development. (Vol. 1). Englewood Cliffs, NJ: Prentice-Hall (1984).

[20] O’Donnell JM Rodgers DL Lee WW Edelson DP Haag J Hamilton MF Structured and Supported Debriefing. Dallas, TX: American Heart Association (2009).

[21] Center for Medical Simulation. Debriefing Assessment for Simulation in Healthcare. Available from: https://harvardmedsim.org/debriefing-assessment-for-simulation-in-healthcare-dash/

[22] Jacobs LM Jr Burns KJ Darko R Development of the medical and surgical simulation institute: Accra Ghana, West Africa. Bull Am Coll Surg (2010) 95(6):23–30. 21452652

